# Estimation of safe zones for suture anchor placement in arthroscopic hip labral repair

**DOI:** 10.3389/fsurg.2026.1681289

**Published:** 2026-03-26

**Authors:** Xinkun Han, Xinyu Tang, Liping Li, Yi Zhang, Haitao Fu, Chao Qi

**Affiliations:** 1 Department of Sports Medicine, The Affiliated Hospital of Qingdao University, Qingdao University, Qingdao, Shandong, China; 2 Medical Department, Qingdao University, Qingdao, Shandong, China; 3 Department of Traumatology, Qingdao Central Hospital, University of Health and Rehabilitation Sciences, Qingdao, Shandong, China

**Keywords:** 3D printing, acetabular, hip arthroscopy, labrum, safe zone

## Abstract

**Background:**

During arthroscopic labral repair, anchors are inserted into the acetabular rim between 10- and 4-o'clock via multiple portals. This study evaluates *in situ* bone quality along each trajectory and precisely defines the spatial relationships between the drill path and adjacent neurovascular, capsular, and chondral structures, thereby establishing an evidence-based safety envelope for acetabular anchor placement.

**Methods:**

In stage one, 11 patients (22 hips) underwent skin marking of surface portals followed by 0.625 mm CT acquisition; Mimics reconstructed the bony model, and Autodesk Maya simulated anchor trajectories to define a preliminary safety zone. In stage two, six formalin-fixed specimens (12 hips) were equipped with 3-D-printed, patient-specific guides after portal marking, dissected, and instrumented at 10–4 o'clock positions. Anchors inserted via anterior, anterolateral, and DALA portals validated the virtual safety map, after which the refined data were applied prospectively in clinical application.

**Results:**

Virtual anchoring revealed marked, position-dependent differences: the DALA portal at 12-o'clock achieved only 50.0% success, significantly below the anterolateral (86.4%) and anterior (90.9%) portals (*P* *=* *0.004*); the anterior portal at 1-o'clock reached 95.5%, exceeding both anterolateral (46.7%) and DALA (27.3%) (*P* *<* *0.001*); DALA at 3-o'clock yielded 77.3%, surpassing anterolateral (27.3%) and anterior (13.6%) (*P* *<* *0.001*); and anterolateral at 4-o'clock fell to 18.2%, well below DALA (86.4%) and anterior (72.7%) (*P* *<* *0.001*). Success rates at 10-, 11-, and 2-o'clock did not differ among portals. Cadaveric validation closely mirrored these trends: no significant inter-portal differences were observed at 10-, 11-, 12-, 1-, 2-, or 3-o'clock, whereas 4-o'clock showed a significant disparity (*χ*² = 8.222, *P* *=* *0.016*), with the anterior portal (75.0%) outperforming the anterolateral (16.7%); DALA (50.0%) did not differ significantly from either. Adherence to these validated trajectories enhances procedural safety and anchor reliability.

**Conclusion:**

For arthroscopic labral repair, anchor placement should follow these portal-specific safe corridors: anterior portal—10, 11, 12, 1, 2, and 4 o'clock; anterolateral portal—10, 11, 12, and 2 o'clock; DALA portal—10, 11, 2, 3, and 4 o'clock.

## Introduction

The acetabular labrum is a C-shaped fibrocartilaginous ring that attaches circumferentially to the acetabular rim. By deepening the socket and enlarging its articular surface, it creates a negative-pressure seal that improves femoral head containment and joint stability (1, 2). The labrum also attenuates peak contact pressures, dissipates shear forces, and minimizes cartilage wear during activities such as walking, running, jumping, and rapid directional changes. Acute torsional trauma, forced hyper-rotation or distraction, chronic repetitive microtrauma, inflammation, and age-related degeneration can all result in labral tears. Such pathology is frequently observed in femoroacetabular impingement syndromes (3). Typical clinical manifestations include anterior, snapping and catching sensations in the hip, in addition to anterior and lateral hip pain, or groin pain. Labral disruption alters hip biomechanics, accelerates degenerative changes, and predisposes to early osteoarthritis (4).

When conservative measures—rest, physiotherapy, and pharmacotherapy—fail to relieve function-limiting symptoms of labral tears, arthroscopic suture repair is indicated (5). The procedure aims to restore labral function, preserve native anatomy, and delay osteoarthritic degeneration, with early intervention reducing the risk of further labral damage and secondary chondral injury (6, 7). Bioabsorbable suture anchors are inserted into the acetabular rim; sutures are passed through the torn labrum and tied to re-approximate the tissue to its anatomic attachment, thereby re-establishing the chondrolabral seal (8, 9). Arthroscopy offers minimal invasiveness, rapid recovery, excellent visualization, and precise instrumentation (10–12).

**Figure 1:**
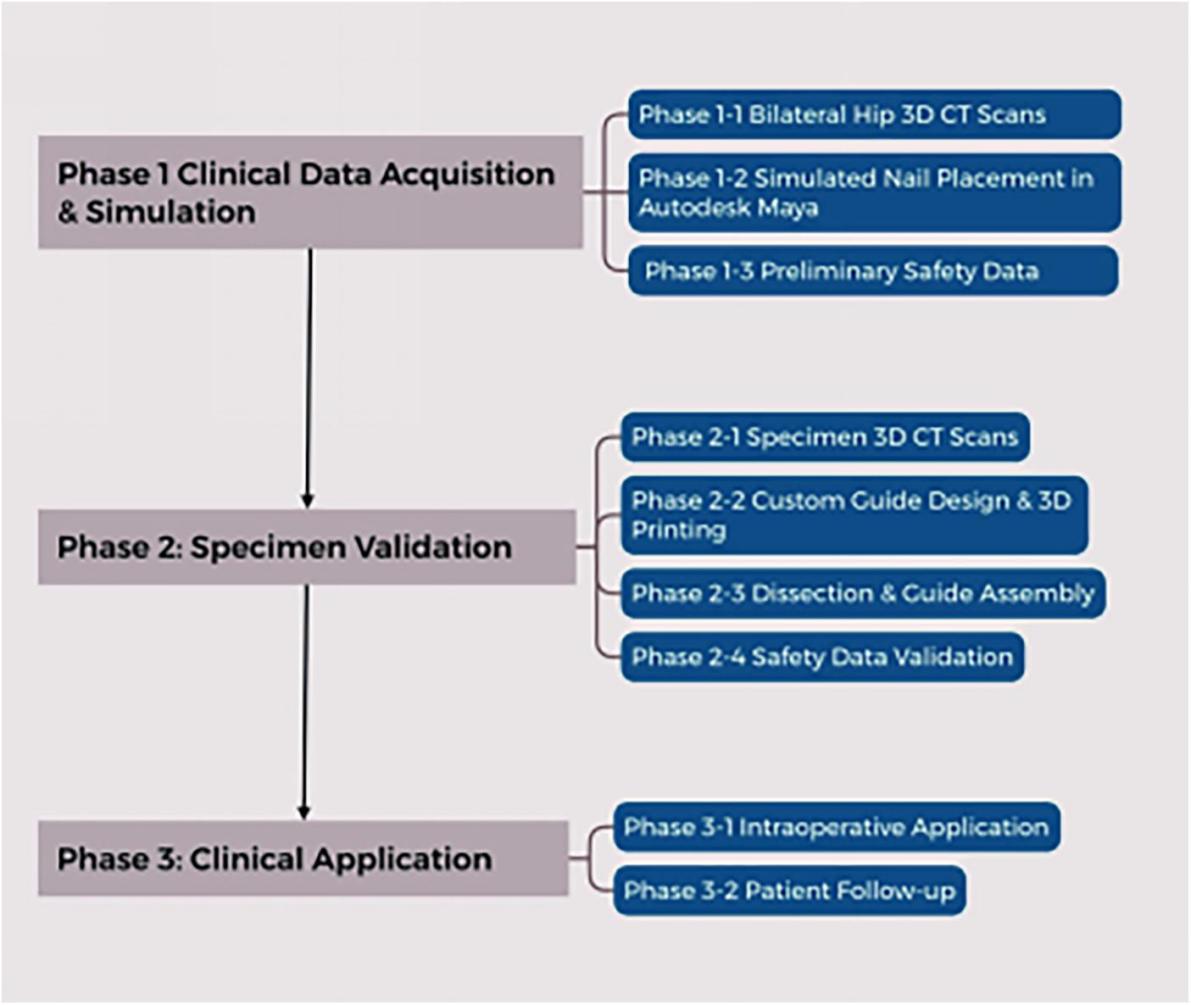
Flowchart for safety zone evaluation of staple path drilling in arthroscopic hip labrum suture.

During acetabular anchor insertion, surgeons routinely assess whether the implant risks damaging adjacent soft-tissue and osseous structures. The procedure is characterized by a narrow margin for error and high potential morbidity; misplacement not only prolongs operative time but also induces irreversible iatrogenic injury to periacetabular tissues, compromises anchor fixation, and adversely affects patient prognosis (13, 14). Despite these risks, no consensus exists regarding the anatomically safe zones for anchor placement. Therefore, this study sought to pre-operatively define optimal insertion sites on the acetabular surface for the most common surgical portals, with the goal of enhancing procedural safety and success rates (Figure 1).

## Materials and methods

The study protocol was reviewed and approved by the Medical Ethics Committee of Qingdao University Affiliated Hospital (QYFY WZLL 30393).Prior to the commencement of the study, all participating patients were fully informed about the study content and voluntarily signed informed consent forms. This study enrolled 11 consecutive patients (8 male, 3 female; age 37–65 years, mean 47 ± 8.2 years) with femoroacetabular impingement syndrome who were treated at Qingdao University Affiliated Hospital. All imaging and demographic data were obtained from the institutional database. The inclusion criteria included: (1) intact osseous and soft-tissue hip anatomy; (2) absence of structural hip deformity; (3) no prior hip fracture or ligament injury; and (4) no previous hip surgery. The exclusion criteria included: (1) severe acetabular or femoral head deformity; (2) active infection, osteonecrosis, or neoplasia of the hip; (3) advanced osteoporosis (T-score ≤ −2.5); and (4) pregnancy or planned pregnancy within 12 months.

## Labeling the patient's surface portals site and three-dimensional reconstruction of the skeleton model

Under fluoroscopic guidance, surface landmarks were drawn on each patient while supine. A radio-opaque marker was placed over the apex of the greater trochanter and a second marker over the anterior-superior iliac spine (ASIS); a distal safety line was then drawn along the long axis of the limb (15). A Kirschner wire (K-wire) was aligned from the ASIS to the greater trochanteric marker, creating an isosceles triangle with the anterolateral and safety lines; the vertex defined the anterior portal. The intersection of the extended ASIS–trochanter line with the superior border of the greater trochanter was designated the DALA (distal anterolateral access) portal (15).

**Figure 2:**
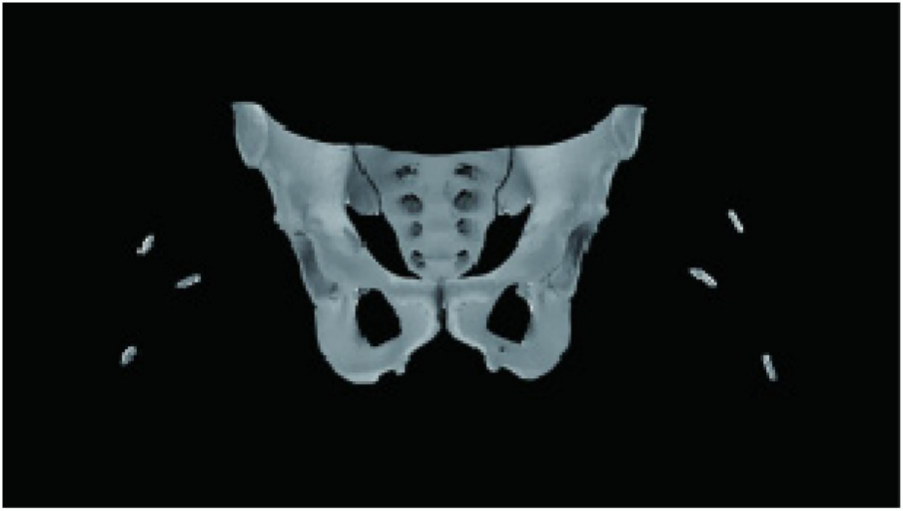
Three-dimensional model image with body surface markers.

Following skin marking, 64-slice helical CT was acquired at 0.625 mm slice thickness. DICOM data were imported into Mimics 21.0 to generate a three-dimensional osseous model that retained the surface markers (Figure 2). The femora were then digitally resected, leaving only the acetabulum and caudal vertebrae for subsequent analysis.

## Three-dimensional simulation nailing

### Three-dimensional reconstruction of suture rivets

**Figure 3:**
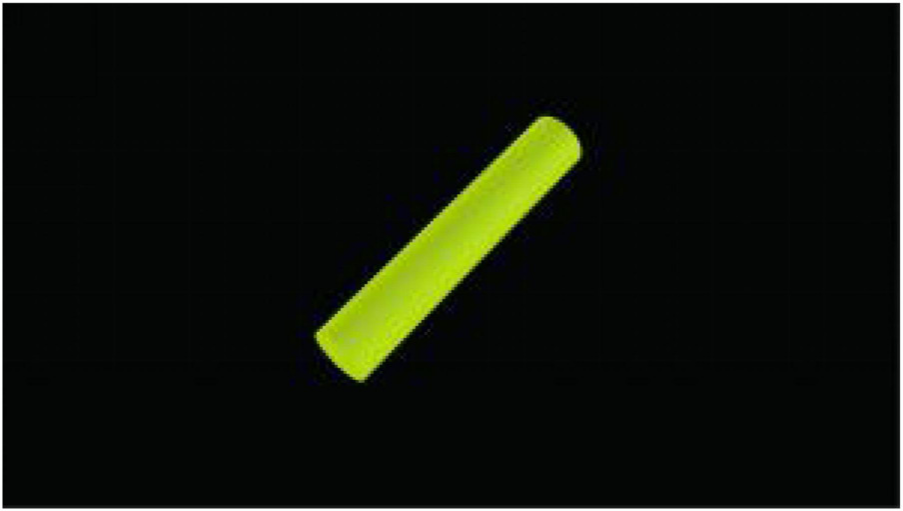
Reconstructed 3.0 mm rivets.

The Arthrex 3.0 mm (USA) suture rivets were reconstructed using Autodesk Maya software (Figure 3). The rivet length (14.5 mm) and diameter (2.7 mm) were all provided by Arthrex Corporation (USA).

### Three-dimensional simulation nailing is carried out in different ways

**Figure 4:**
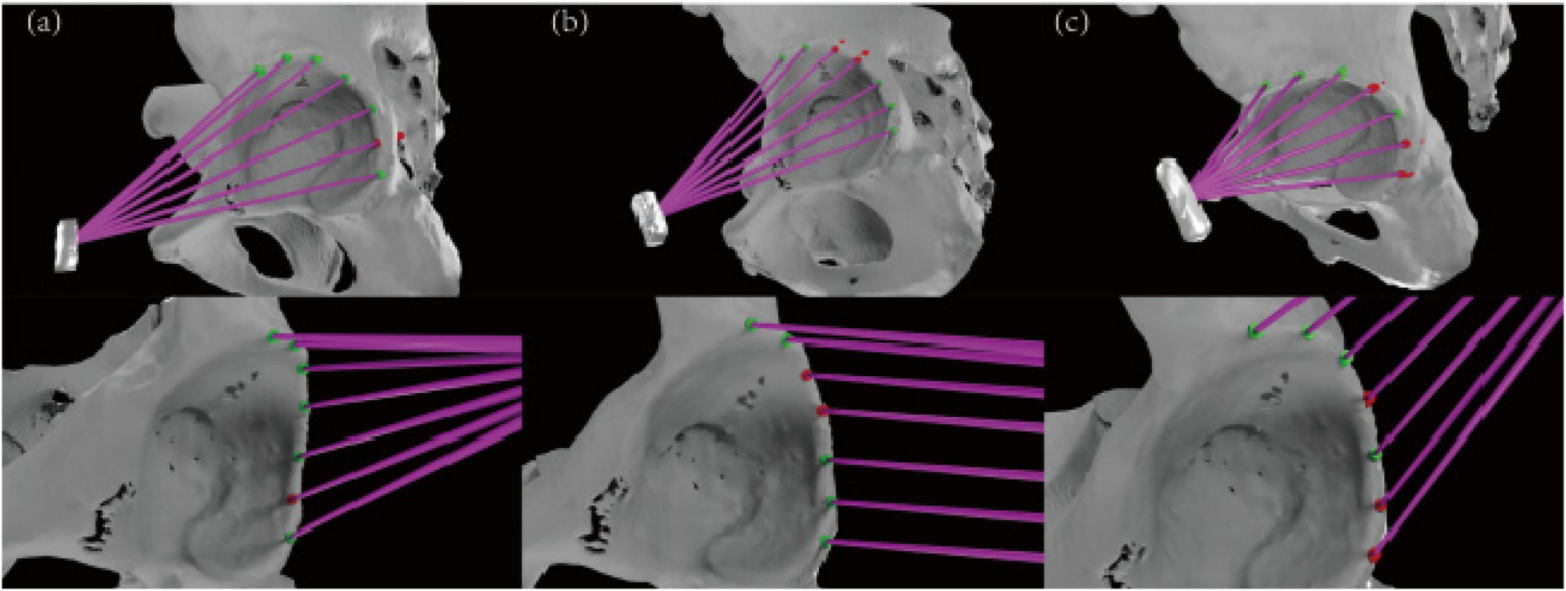
We can observe that when rivets are placed in anterior portal, the placement is only at 3 o'clock in the acetabulum (red) in **(a)**. When rivets are placed in the DALA portal, the placement at 12/1 o'clock in the acetabulum is invalid (red), and the rivets are completely placed in the 10/11/2/3/4 o'clock direction (green) in **(b)**.When the rivet is inserted in anterolateral portal, the placement of the acetabulum at 1/3/4 o'clock is invalid (red), and the rivet is completely inserted on the 10/11/12/2 o'clock (green) in **(c)**.

In Autodesk Maya, A trajectory was defined as “success” (safe) and marked green if the entire suture anchor was contained within the cortical and cancellous bone of the acetabular rim without any breach of the outer cortex. A trajectory was defined as “failure” (unsafe) and marked red if any part of the anchor penetrated the outer cortex, indicating a risk of damaging surrounding neurovascular or soft-tissue structures (Figure 4).

## Hip specimen nailing

Phase I of the three-dimensional simulation yielded the following optimal anchor sites: anterior portal—three safe trajectories; DALA portal—one safe trajectory at the 12- to 1-o'clock position; anterolateral portal—three safe trajectories at the 1-, 3- and 4-o'clock positions. The success rates of these trajectories differed significantly (*P* < 0.05). Because the virtual model did not account for bone quality or intra-operative variables, and because periacetabular soft tissues critically influence anchor stability and clinical outcome, Phase II validated these findings using fresh-frozen human cadaveric hip specimens. Direct implantation allowed precise macroscopic and radiographic assessment of the previously defined “safe” trajectories.

## Anatomy of hip specimens

Six formalin-fixed human cadaveric hemipelves (12 acetabula) were obtained from the Department of Anatomy and Research, Qingdao University. Donors comprised four males and two females aged 41–71 years (mean 51 ± 9.4 years). The inclusion criteria included: (1) intact hip anatomy; (2) absence of structural deformity; (3) no prior hip fracture or ligamentous injury; and (4) no previous hip surgery. The exclusion criteria included: (1) severe acetabular or femoral head deformity; (2) active infection, osteonecrosis, or neoplasia of the hip; and (3) advanced osteoporosis (T-score ≤ −2.5).

Each specimen was transected 25 cm distal to the lumbosacral junction and 20 cm distal to the femoral head–neck junction. Skin, subcutaneous fascia, and all peri-articular muscles and ligaments were left intact. Because no major neurovascular structures traverse the operative field (16), no additional protection was required during dissectioStepwise exposure proceeded as follows: skin and subcutaneous tissue were reflected; the iliopsoas, sartorius, gluteus medius, gluteus minimus, and tensor fasciae latae were sharply dissected and retracted; the hip capsule—originating at the acetabular rim and transverse acetabular ligament and inserting anterior to the intertrochanteric line and posterior to the medial aspect of the intertrochanteric crest—was excised *in toto*. The femur was disarticulated, the labrum was removed, and the sublabral bone (i.e., the intended anchor zone) was fully exposed.

**Figure 5:**
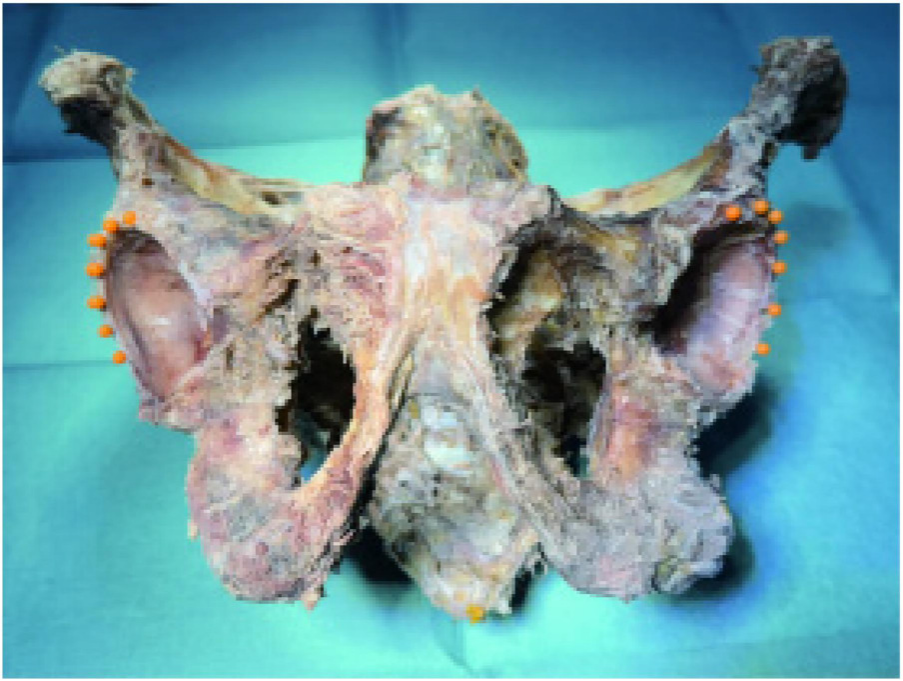
Double-sided pushpin marking rivet placement position.

**Figure 6:**
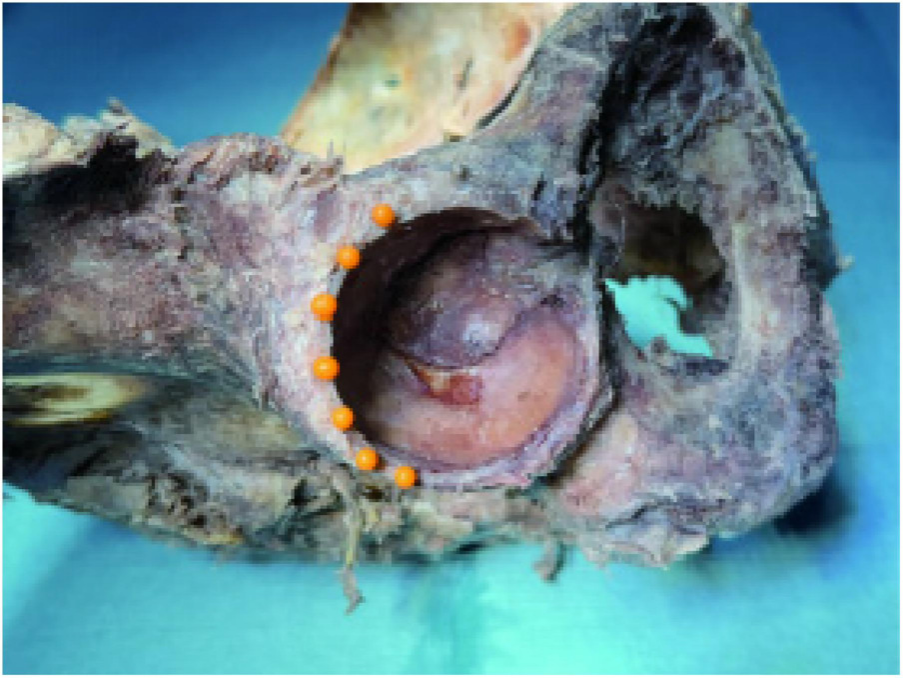
Single-sided pushpin mark rivet placement position.

Seven anchor sites were marked with micro-pins at 2 mm intervals along the acetabular rim from the 10- to 4-o'clock positions, using the medial border of the anterior–inferior iliac spine as the 12-o'clock reference (Figures 5, 6).

## Design and print the body surface access locator

**Figure 7:**
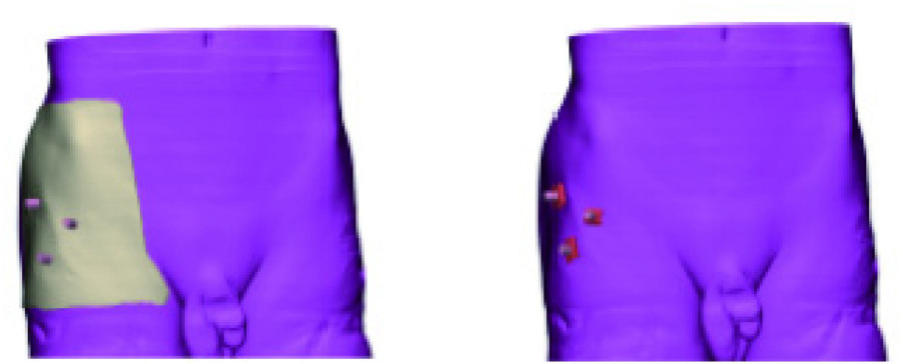
Reconstructed body surface markers; reconstructed skin.

**Figure 8:**
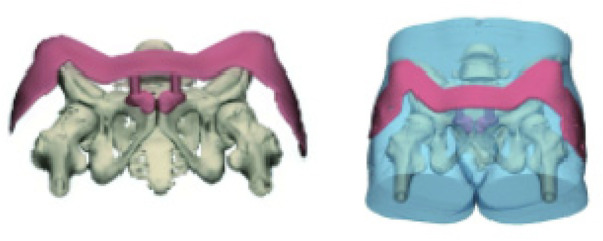
The pubic junction extends to the skin position; design the entry position according to the skin.

**Figure 9:**
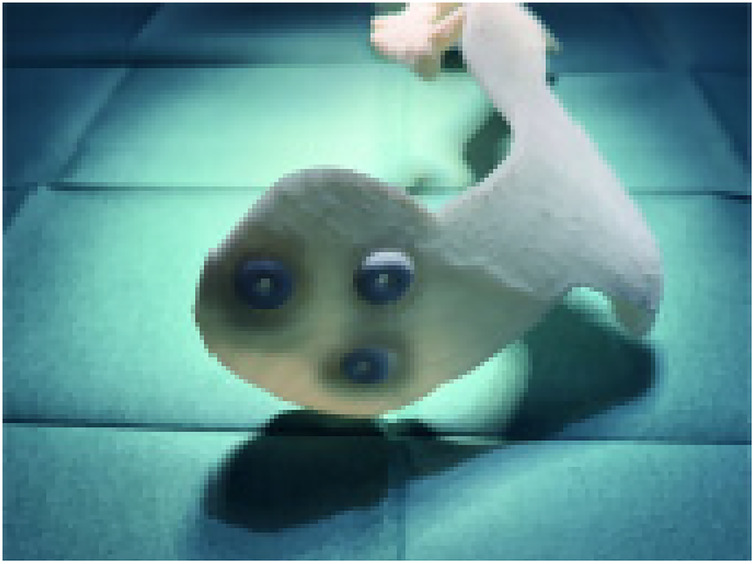
3D printing and filled body surface access positioning plate.

**Figure 10:**
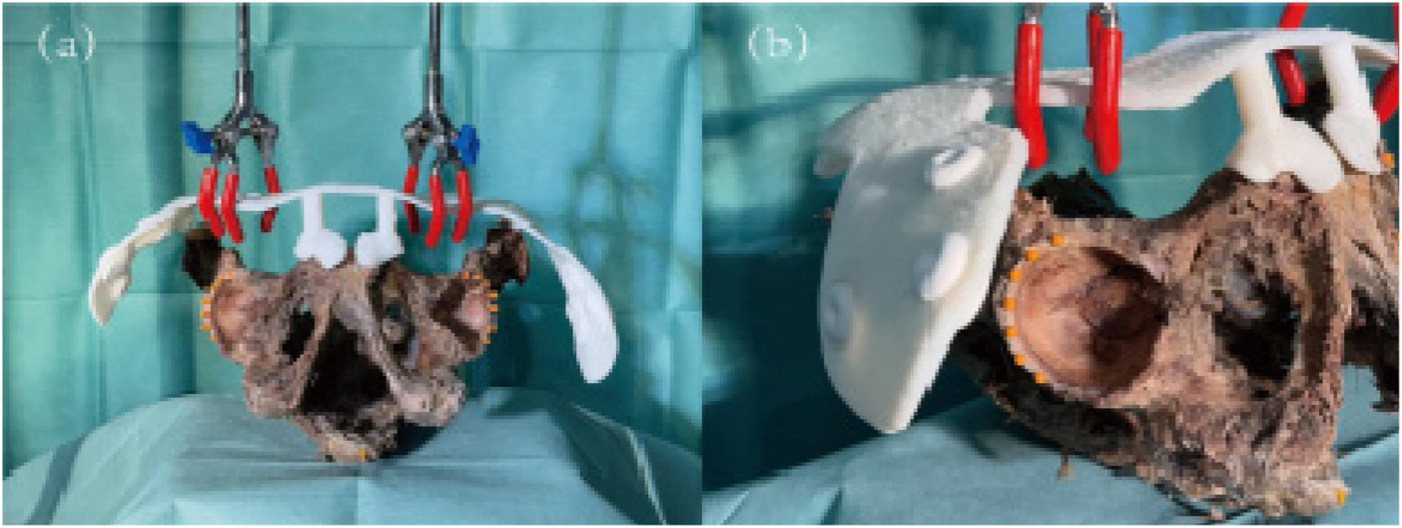
(**a**) Frontal view of positioning plate and specimen assembly; (**b**) side view of positioning plate and specimen assembly.

Due to the limitations of formalin fixation for intraoperative traction, this study employed an open anatomical approach. To preserve surface landmarks, all specimens were marked according to clinical standards before dissection and underwent CT scanning (slice thickness: 0.625 mm). After acquiring DICOM data, a personalized guide was designed through 3D reconstruction and molded along the pubic symphysis axis (Figures 7, 8). The guide was fabricated using acrylonitrile butadiene styrene (ABS) material via the fused deposition modeling (FDM) process, offering favorable mechanical properties. Access channels were filled with hard modeling clay (Figure 9) to simulate soft tissue resistance, enabling accurate repositioning of skin portals post-resection. The guide was secured to the pubic symphysis using custom clamps and tripod forceps (Figure 10), replicating the clinical traction position. Adaptation accuracy was ensured by verifying complete contact with bony landmarks and alignment of the drill sleeve orientation with the virtual plan.

## Simulated surgical suture anchor placement

**Figure 11:**
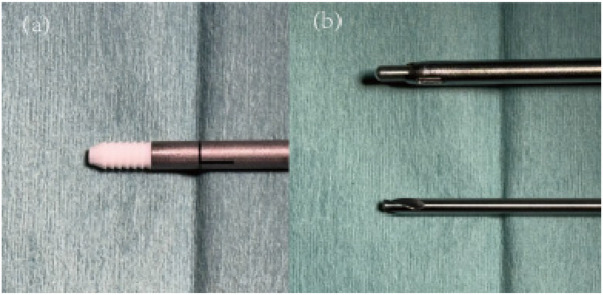
(**a**) 3.0 mm rivets; (**b**) nail opening and drill bit.

**Figure 12:**
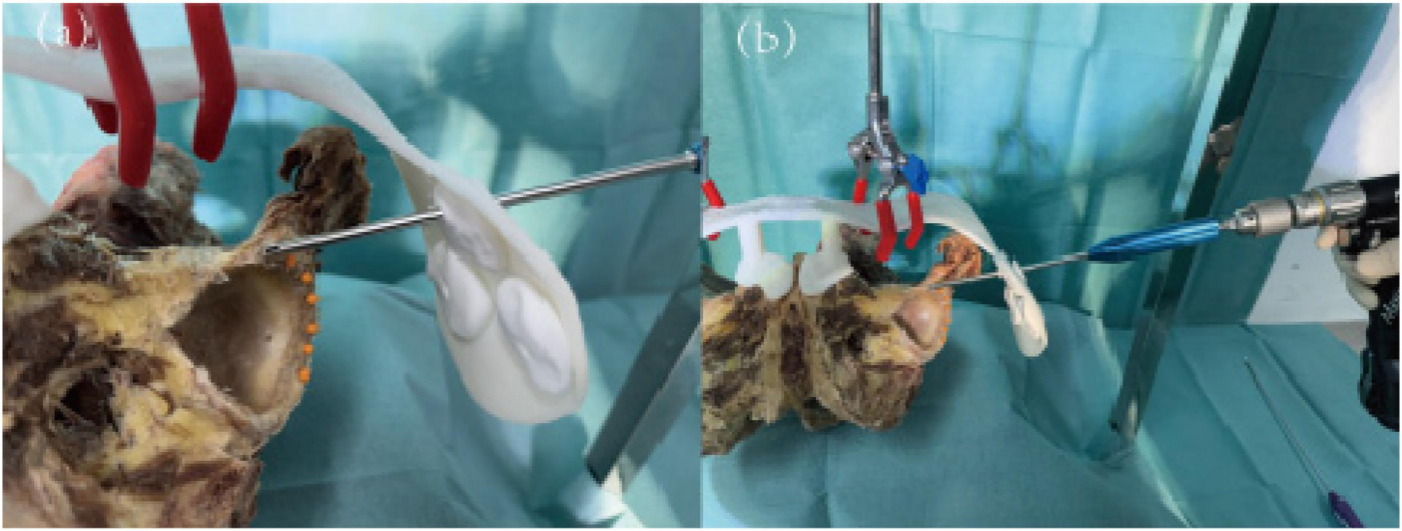
(**a**) Positioning of nail path opening; (**b**) nail path drilling.

Following anatomical preparation, anchors were inserted through the anterior, DALA, and anterolateral portals into the seven pre-marked acetabular sites. A 3.0 mm Arthrex suture anchor (Figure 11a) was delivered via the 3-D-printed surface locator through a 2.3 mm skin incision (Figure 11b). The anterolateral portal was subsequently used to access the same seven sites (Figure 12). Each insertion was video-documented, with particular attention to periacetabular soft-tissue injury and post-insertion anchor stability, thereby providing safety benchmarks for arthroscopic labral repair and validating the virtual data obtained in Phase I.

**Figure 13:**
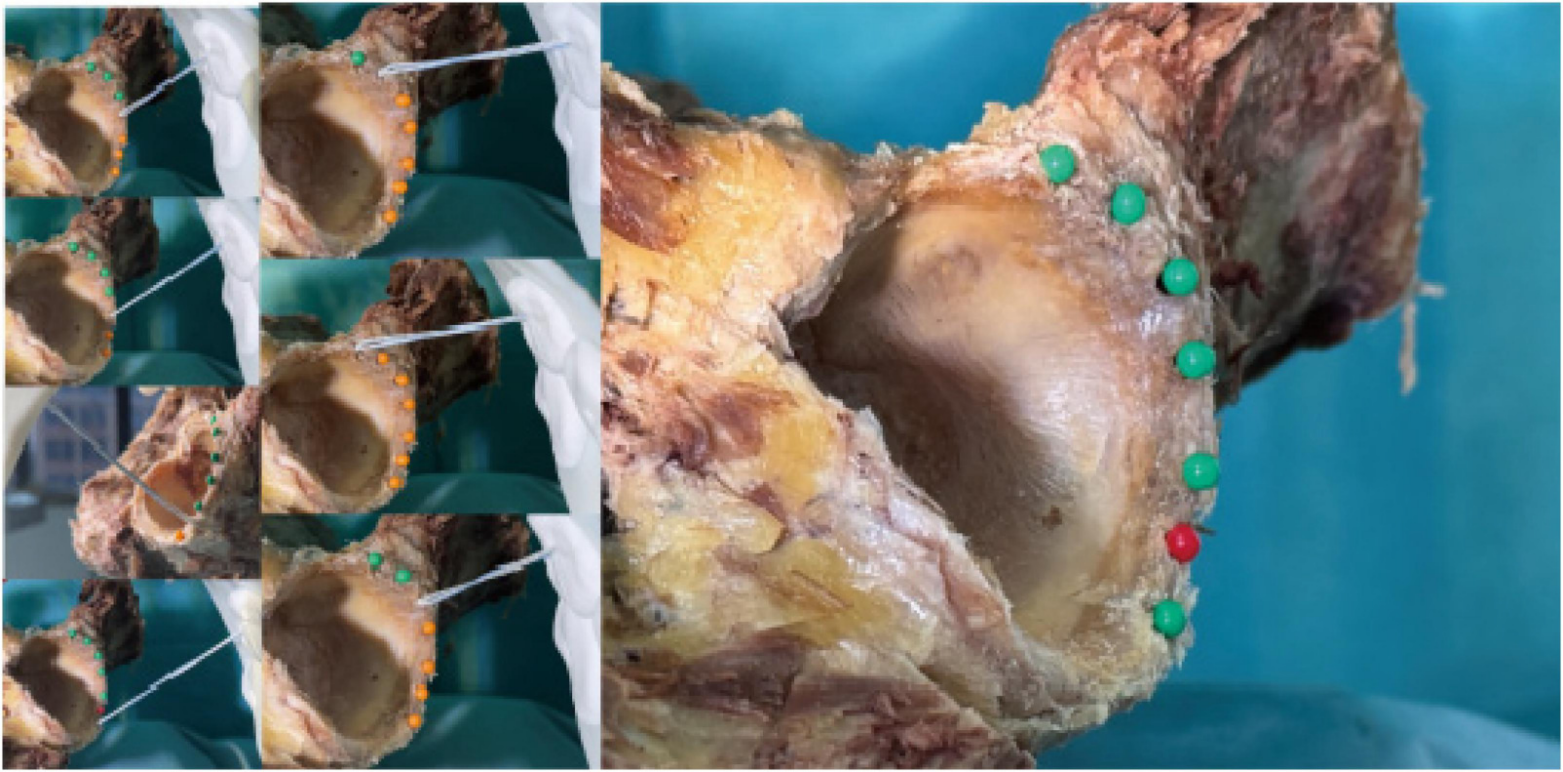
The situation of nailing in anterior portal; results of anterior portal nailing.

**Figure 14:**
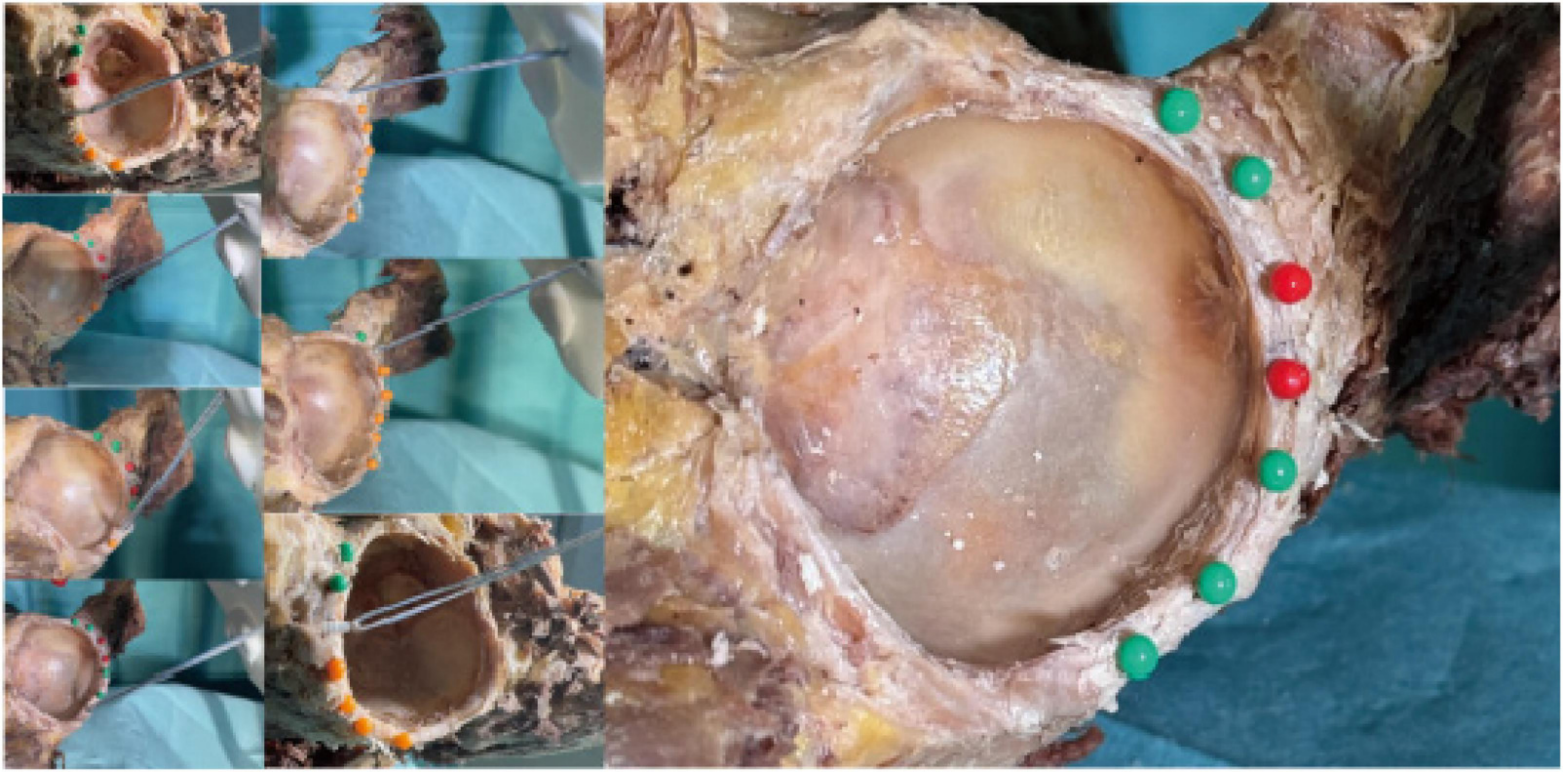
The situation of nailing in DALA portal; results of DALA portal nailing.

**Figure 15:**
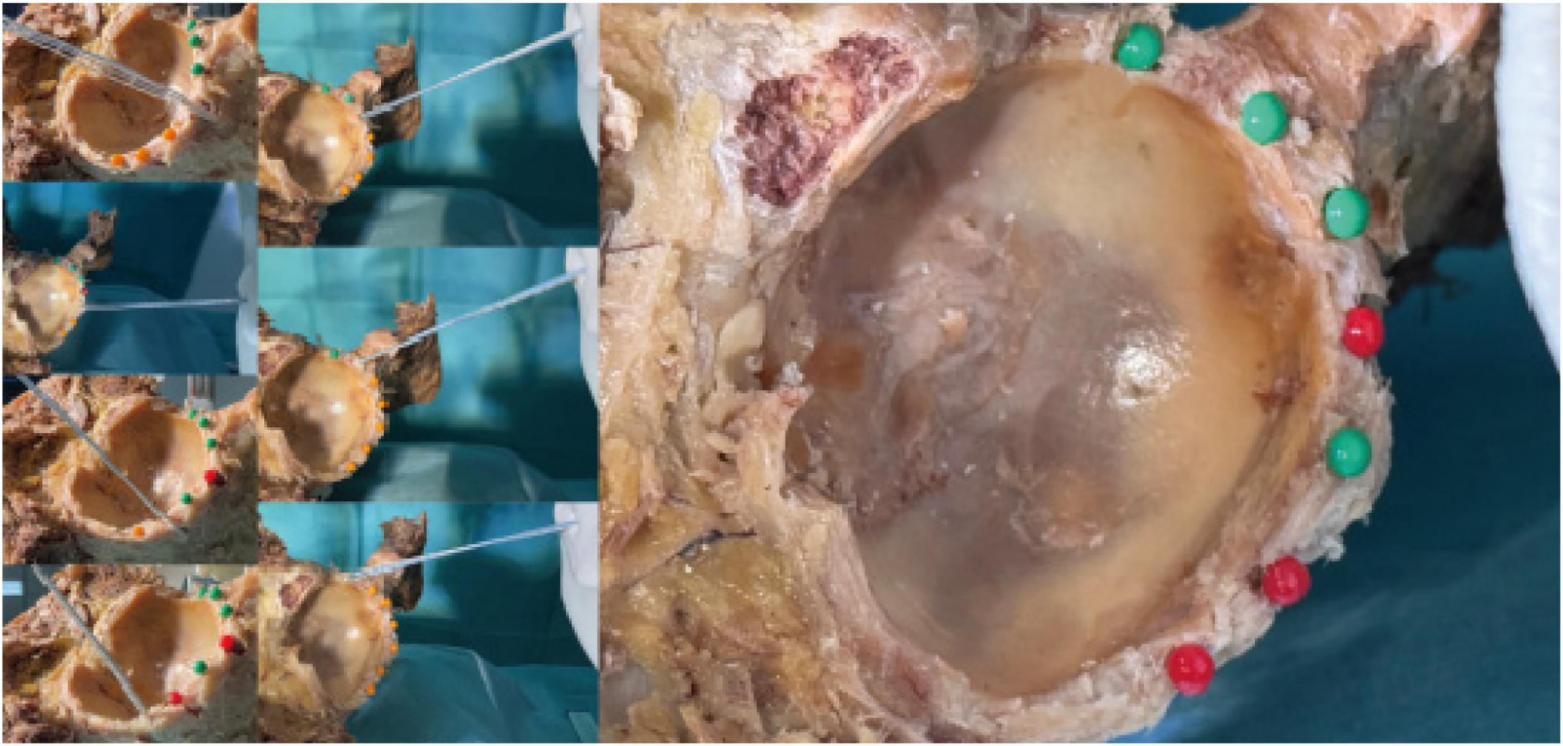
The situation of nailing in anterolateral portal; results of anterolateral portal nailing.

Following anchor insertion, the high-strength suture was oriented parallel to the anchor axis and exited smoothly through the centre of the skin portal. A site was considered safe—marked with a green pin—when the anchor lay entirely within bone and caused no visible damage to periacetabular structures. Sites associated with cortical perforation or soft-tissue injury were deemed hazardous and marked with a red pin (Figures 13–15).

## Result

### Data statistics on three-dimensional simulation nailing stage

Phase-I virtual anchoring revealed marked site-dependent differences in success rates across the acetabular clock-face. Cochran's *Q* test and Dunn's test (with Bonferroni correction) were used to demonstrate the differences.Success rates at 10- and 11-o'clock were comparable among all portals (86.4%–95.5%; *P* = 0.549 and *P* = 0.819, respectively). At 12-o'clock, the DALA portal achieved only 50.0%, significantly lower than both the anterolateral (86.4%) and anterior (90.9%) portals (*P* = 0.004). Conversely, the anterior portal at 1-o'clock reached 95.5%, surpassing the anterolateral (46.7%) and DALA (2-o'clock, 81.8%) portals (*P* < 0.001). At 2-o'clock, all portals performed similarly (81.8%–86.4%; *P* = 0.950). At 3-o'clock, DALA yielded 77.3%, exceeding the anterolateral (27.3%) and anterior (13.6%) portals (*P* < 0.001). Finally, at 4-o'clock, the anterolateral portal dropped to 18.2%, markedly below DALA (86.4%) and anterior (72.7%) portals (*P* < 0.001). These findings underscore that portal selection is the primary determinant of anchor success at positions 12-, 1-, 3-, and 4-o'clock (Tables 1–3).

**Table 1:** Nail setting in three-dimensional simulation stage.

Goal Type	Acetabular Point	Total number of nailing times	Number of Success	Number of failures	Success rate (%)
Anterior portal	10	22	19	3	86.4
	11	22	20	2	**90** **.** **9**
	12	22	20	2	**90**.**9**
	1	22	21	1	**95**.**5**
	2	22	18	4	81.8
	3	22	3	19	**13**.**6**
	4	22	16	6	72.7
Anterolateral portal	10	22	20	2	**90**.**9**
	11	22	20	2	**90**.**9**
	12	22	19	3	86.4
	1	22	7	15	**46**.**7**
	2	22	18	4	81.8
	3	22	6	16	**27**.**3**
	4	22	4	18	**18**.**2**
DALA portal	10	22	21	1	**95**.**5**
	11	22	21	1	**95**.**5**
	12	22	11	11	**50**.**0**
	1	22	6	16	**27**.**3**
	2	22	19	3	86.4
	3	22	17	5	77.3
	4	22	19	3	86.4

Bolded data represents the extreme values at both ends.

**Table 2:** Comparison of the differences in different portals of different acetabular sites in three-dimensional simulation experiments (Cochran's *Q* test).

Acetabular point	DALA portal	Anterolateral portal	Anterior portal	*Χ* ^2^	*P*
10 o'clock				1.200	0.549
Success	21	20	19		
Fail	1	2	3		
11 o'clock				0.400	0.819
Success	21	20	20		
Fail	1	2	2		
12 o'clock				11.231	0.004
Success	6	7	21		
Fail	16	15	1		
1 o'clock				22.211	<0.001
Success	6	7	21		
Fail	16	15	1		
2 o'clock				0.200	0.950
Success	19	18	18		
Fail	3	4	4		
3 o'clock				18.111	<0.001
Success	17	6	3		
Fail	5	16	19		
4 o'clock				21.000	<0.001
Success	19	4	16		
Fail	3	18	6		

**Table 3:** Differential comparisons of different portals in different acetabular sites in three-dimensional simulation experiments [(Dunn's test (corrected by Bonferroni method)].

Acetabular point	Way to portal 1	Ways to portal 2	*P*
12 o'clock	Anterior portal	Anterolateral portal	>0.999
	Anterior portal	DALA portal	0.007
	Anterolateral portal	DALA portal	0.020
1 o'clock	Anterior portal	Anterolateral portal	<0.001
	Anterior portal	DALA portal	<0.001
	Anterolateral portal	DALA portal	>0.999
3 o'clock	Anterior portal	Anterolateral portal	>0.999
	Anterolateral portal	DALA portal	0.004
	Anterior portal	DALA portal	<0.001
4 o'clock	Anterior portal	DALA portal	>0.999
	Anterolateral portal	DALA portal	<0.001
	Anterior portal	Anterolateral portal	<0.001

The Dunn's test, coupled with Bonferroni correction for all pairwise comparisons, was applied. The adjusted significance level was set at *α*′ = 0.05/21 ≈ 0.0024, with *P* < 0.0024 denoting statistical significance.

### Statistics of the data of specimen nailing stage

Cadaveric testing revealed no significant inter-portal differences at 10-, 11-, or 12-o'clock (*P* = 0.368, 0.417, 0.150), with success rates spanning 83.3%–91.7%, 58.3%–83.3%, and 41.7%–83.3%, respectively. At 1-o'clock the anterior portal achieved 75.0% success compared with 33.3% for DALA and anterolateral portals, although the difference did not reach significance (*P* = 0.062). Similarly, no significant disparities were observed among the three portals at 2- or 3-o'clock positions. A significant difference emerged only at the 4-o'clock position (*P* = 0.016), where the anterior portal's 75.0% success rate exceeded the anterolateral portal's 16.7%, while the DALA portal's 50.0% did not differ significantly from either comparator; no significant differences were observed at the other clock positions.In this phase, Cochran's *Q* test along with Dunn's test (with Bonferroni correction) were again employed.Overall, the rank order of success rates across the acetabular clock-face closely matched the three-dimensional simulation, confirming the predictive validity of the virtual safety map (Tables 4–6).

**Table 4:** Nail placement in the specimen stage.

Goal Type	Acetabular Point	Total number of nailing times	Number of Success	Number of failures	Success rate (%)
Anterior portal	10	12	11	1	**91** **.** **7**
	11	12	10	2	83.3
	12	12	10	2	83.3
	1	12	9	3	75.0
	2	12	10	2	83.3
	3	12	5	7	**41**.**7**
	4	12	9	3	75.0
Anterolateral portal	10	12	8	4	66.7
	11	12	7	5	58.3
	12	12	7	5	58.3
	1	12	4	8	**33**.**3**
	2	12	6	6	**50**.**0**
	3	12	3	9	**25**.**0**
	4	12	2	10	**16**.**7**
DALA portal	10	12	10	2	83.3
	11	12	9	3	75.0
	12	12	5	7	**41**.**7**
	1	12	4	8	**33**.**3**
	2	12	8	4	66.7
	3	12	7	5	58.3
	4	12	6	6	**50**.**0**

Bolded data represents the extreme values at both ends.

**Table 5:** Comparison of the differences in different portals in different acetabular sites in the verification of specimen.

Acetabular point	DALA portal	Anterolateral portal	Anterior portal	*Χ* ^2^	*P*
10 o'clock				2.000	0.368
Success	10	8	11		
Fail	2	4	1		
11 o'clock				1.750	0.417
Success	9	7	10		
Fail	3	5	2		
12 o'clock				3.800	0.150
Success	5	7	10		
Fail	7	5	2		
1 o'clock				5.556	0.062
Success	4	4	9		
Fail	8	8	3		
2 o'clock				2.667	0.264
Success	8	6	10		
Fail	4	6	2		
3 o'clock				2.667	0.264
Success	7	3	5		
Fail	5	9	7		
4 o'clock				8.222	0.016
Success	6	2	9		
Fail	6	10	3		

**Table 6:** Pairwise comparison of different portals at different acetabular sites in specimen verification [(Dunn's test (corrected by Bonferroni method)].

Acetabular point	Way to portal 1	Ways to portal 2	*P*
4 o'clock	Anterior portal	DALA portal	0.662
	Anterior portal	Anterolateral portal	0.013
	Anterolateral portal	DALA portal	0.307

### Safety data on clinical application

Integrating the two experimental phases, this study provides evidence-based guidance for the selection of surgical portals and anchor placement sites in arthroscopic labral repair. The anterior portal should avoid anchor placement at the 3 o'clock position; all other sites are considered safe. The DALA portal requires particular caution at the 12 and 1 o'clock positions. Whenever possible, it is advisable to restrict anchor placement via the anterolateral portal to sites other than the 1, 3, and 4 o'clock positions.

To clinically validate these safety data, we applied the experimental findings to perform arthroscopic hip labral repair in 27 patients, with 19 of them undergoing a six-month follow-up. Detailed records were kept regarding the number of drill attempts for anchor placement and the total surgical duration. All patients received comprehensive preoperative informed consent. Under general anesthesia, each patient was positioned supine on a traction table to optimize traction control and fluoroscopic imaging. A well-padded perineal post was placed to reduce the risk of pudendal nerve injury (17). Traction was first applied to the contralateral limb with the hip abducted at 45°; the affected limb was then gently adducted and internally rotated (18). The traction force was maintained below 34 kg, with a traction duration of less than 2 h, achieving a joint distraction of 8–10 mm (1, 19).

**Figure 16:**
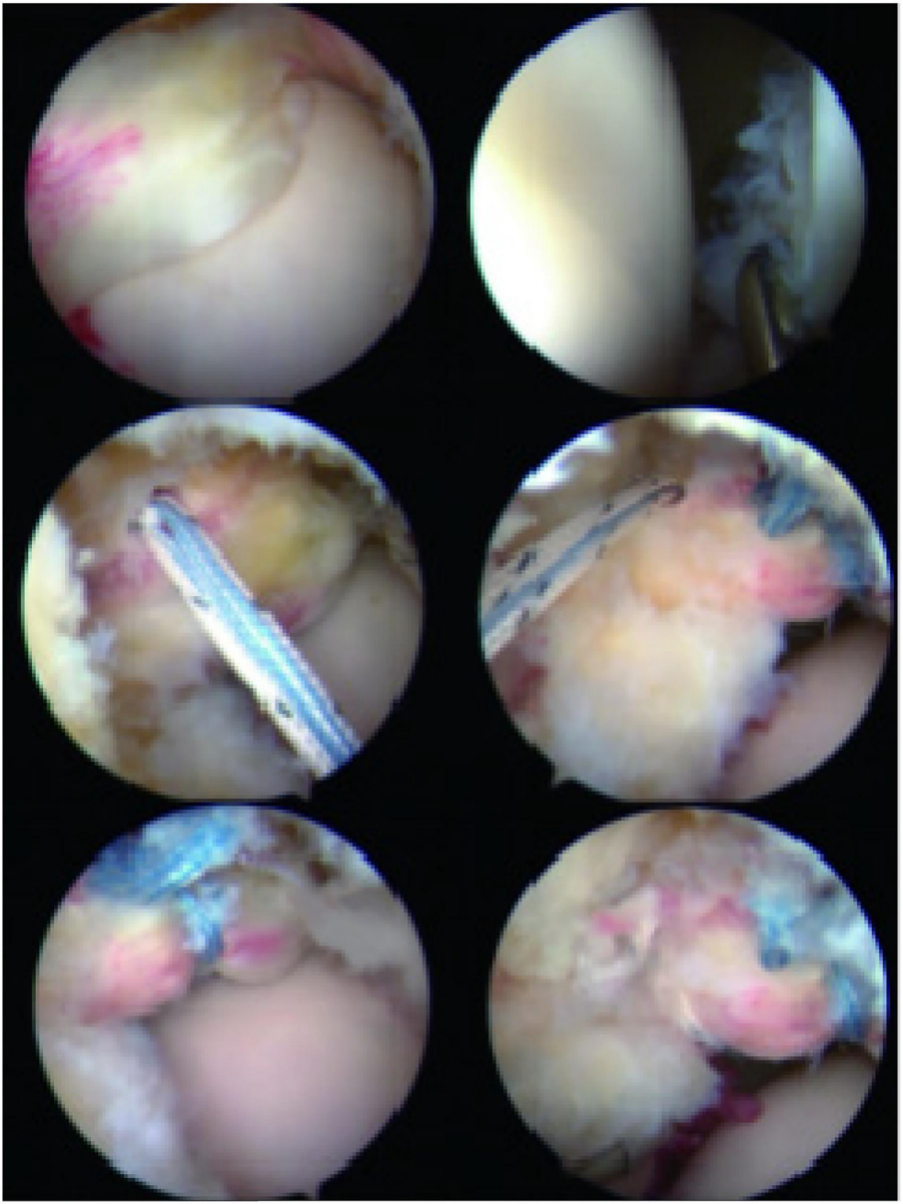
Intraoperative nail suture process.

Surgical procedures utilized the experimentally derived safety data for the anterior, DALA, and anterolateral portals. Direct arthroscopic visualization served as the gold standard for diagnosing labral tears (Figure 16), allowing precise identification of tear geometry and guiding accurate anchor placement based on the predefined safety criteria (20).

The locations of labral tears observed arthroscopically in the 19 patients who underwent follow-up ranged from the 10 o'clock to the 4 o'clock positions. Detailed records were kept for each patient based on age, sex, affected side, BMI, number of anchor drillings, operative time, preoperative HHS, and postoperative HHS. In our previous follow-up records of patients who underwent labral repair surgery, we compared the clinical outcomes before and after the application of the experimental conclusions (Table 7).

**Table 7:** Comparative baseline characteristics of the two patient groups.

Item	Before Implementation of Conclusions	After Implementation of Conclusions	Statistical Value	*P*
Age (years, $\bar{x}\pm s$)	33.6 ± 10.1	33.0 ± 8.8	0.076	0.964
Gender (Male/Female, n)	12/7	9/10	0.461	0.872
Affected Side (Left/Right, n)	8/11	10/9	0.708	0.783
BMI(kg/m^2^, n)	26.7 ± 5.4	24.8 ± 4.3	2.834	0.114

**Figure 17:**
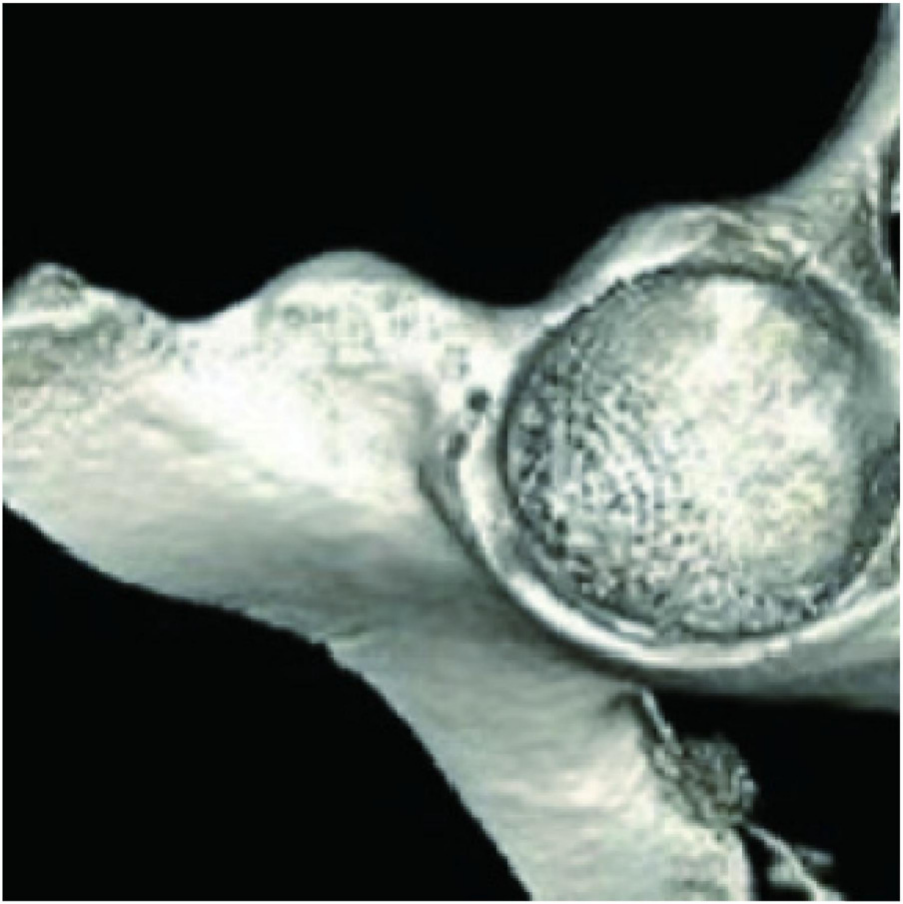
Postoperative three-dimensional CT.

A comparison between the two groups revealed no statistically significant differences in the baseline characteristics of the patients in each group *(P* *>* *0.05*). Applying the experimental findings significantly shortened the surgical duration compared to the pre-implementation cohort (*P* *<* *0.001*) and markedly reduced the number of drill attempts for anchor placement (*P* *<* *0.001*). Postoperative three-dimensional CT imaging confirmed that all anchor trajectories were completely within the bone (Figure 17). Preoperative Harris Hip Scores (HHS) showed no significant difference between the pre-implementation and post-implementation groups (58.4 ± 6.9 vs. 56.7 ± 3.8, *P* = 0.141). Postoperatively, both groups demonstrated significant improvements from baseline (both *P* < 0.001). However, the magnitude of improvement was notably greater in the post-implementation group, with a mean increase of 33.3 points compared to 28.7 points in the pre-implementation group. The post-implementation group also achieved significantly higher postoperative HHS scores (90.0 ± 3.4 vs. 87.1 ± 4.0, *P* = 0.033) (Table 8). These results demonstrate that the conclusions derived from this study have a meaningful impact on arthroscopic hip labral repair, notably enhancing the success rate of anchor placement while substantially reducing operative time.

**Table 8:** Comparative analysis of the two patient groups before and after the implementation of conclusions.

Item	Before Implementation of Conclusions	After Implementation of Conclusions	*F*	*P*
Preoperative HHS Score	58.4 ± 6.9	56.7 ± 3.8	1.897	0.141
Postoperative HHS Score	87.1 ± 4.0	90.0 ± 3.4	3.687	0.033
*F*	108.728	129.823		
*P*	**<0.001**	**<0.001**		
Opration Time	94.3 ± 17.6	78.4 ± 8.7	54.652	**<0**.**001**
Number of Suture Anchors	4.2 ± 0.9	2.7 ± 0.7	65.77	**<0**.**001**

*P*-values in bold indicate statistical significance at a significance level of 0.05.

## Discussion

Over the nine decades since Burman's pioneering arthroscopic examination of a cadaveric hip in 1931 and his first diagnostic procedure in 1935 (21), hip arthroscopy has matured into the cornerstone of modern joint-preserving surgery. The present study introduces an innovative, data-enriched surgical strategy for labral repair, characterized by its integration of detailed 3D preoperative planning with rigorously validated, portal-specific safety profiles. This methodology establishes a systematic and reproducible clinical framework that empowers surgeons to optimize anchor placement accuracy and enhance postoperative outcomes.

Acetabular labral injuries can be addressed through various hip arthroscopic procedures such as labral repair, debridement, augmentation, and reconstruction (22).

Arthroscopic labral augmentation and debridement are technically straightforward and minimally invasive procedures. However, they carry a risk of excessive resection of healthy labral tissue. While labral debridement can improve patients' range of motion and alleviate pain, nearly one-third of these patients subsequently undergo total hip arthroplasty (THA) (23, 24). This outcome is considered to be associated with the accelerated degenerative changes resulting from over-resection of normal labral tissue. For patients with severe labral damage, labral reconstruction using autograft or allograft tendons may be employed. However, the field of hip labral reconstruction currently requires further research regarding graft selection and clear indications. Moreover, the reconstructed acetabular labrum lacks a blood supply, and its long-term durability remains unknown (25, 26).

Labral repair remains the cornerstone clinical treatment for labral tears. This technique involves re-establishing the normal anatomical position of the torn labrum using suture anchors. Excellent clinical outcomes are achieved by combining this labral repair with arthroscopic resection of bony impingements.

The current surgical procedure involves the following steps: arthroscopic identification of the labral tear site, debridement of the posterior labral recess and adjacent capsular tissue, use of radiofrequency ablation to expose the sublabral bone, placement of suture anchors, and a final inspection to ensure no iatrogenic damage to the periacetabular structures. Furthermore, the creation of anchor tunnels still requires fluoroscopic guidance to prevent intra-articular penetration or breach of the acetabular bone. This technique has a low margin for error and carries significant risks. Anchor placement failure not only creates new iatrogenic injuries for the patient but also prolongs the operative time.

Currently, the selection of anchor trajectories remains an uninvestigated area in the literature. This study introduces a novel strategy for labral repair by establishing optimal anchor placement positions. Our findings indicate that while all three commonly used hip arthroscopy portals (anterior, DALA, and anterolateral) permit anchor placement along the acetabular rim, each portal carries specific risk zones. For the anterior portal, the only hazardous position is at the 3 o'clock direction, with all other positions demonstrating high safety. The DALA portal requires particular caution to avoid anchor placement toward the 12 and 1 o'clock positions, while the anterolateral portal should avoid placement at the 1, 3, and 4 o'clock directions. These precise recommendations effectively minimize the risk of anchor placement failure. Consequently, we recommend the routine clinical use of the anterior portal for anchor insertion, as it offers the largest safe zone and the highest success rate. Our approach enables surgeons to preoperatively determine appropriate superficial pathways and identify safe acetabular anchor sites, significantly improving placement success rates and reducing surgical time.

Nevertheless, this study has several limitations. During the experiments, we did not identify any position that guaranteed 100% successful anchor placement. Although the anchor placement procedures were performed by two collaborating clinicians, the potential for visual and technical errors could not be entirely eliminated. Additionally, the limited number of formalin-preserved cadaveric specimens utilized represents another constraint. While patient-specific guide templates were designed for each specimen, the potential alteration of bone quality due to formalin preservation must be acknowledged. Future studies utilizing fresh-frozen specimens would allow more accurate evaluation of anchor placement efficacy. Furthermore, as our postoperative follow-up cohort expands, the safety profile for anchor placement is expected to become more clearly defined.

## Conclusion

**Figure 18:**
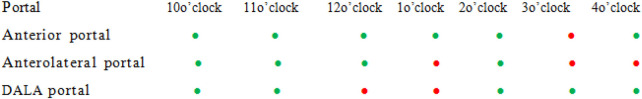
Results of suture anchor insertion via three surgical portals.

During arthroscopic labral repair, inaccurate anchor placement risks iatrogenic injury to periacetabular structures and compromises fixation, ultimately impairing patient outcome. To minimize failure, we recommend the following trajectories: (1) anterior portal—10, 11, 12, 1, 2, and 4 o'clock; (2) anterolateral portal—10, 11, 12, and 2 o'clock; and (3) DALA portal—10, 11, 2, 3, and 4 o'clock (Figure 18). These evidence-based guidelines enhance surgical safety and success rates for labral suture anchor insertion.

## Data Availability

The original contributions presented in the study are included in the article/Supplementary Material, further inquiries can be directed to the corresponding author.
